# Comparison of the accuracy of Hybrid Capture II and polymerase chain reaction in detecting clinically important cervical dysplasia: a systematic review and meta-analysis

**DOI:** 10.1002/cam4.83

**Published:** 2013-04-21

**Authors:** Hung N Luu, Kristina R Dahlstrom, Patricia Dolan Mullen, Helena M VonVille, Michael E Scheurer

**Affiliations:** 1 Dan L. Duncan Cancer Center, Baylor College of MedicineHouston, Texas; 2 Division of Epidemiology, Human Genetics and Environmental Sciences, School of Public Health, University of Texas Health Science Center-HoustonHouston, Texas; 3 Division of Health Promotion and Behavioral Sciences, School of Public Health, University of Texas Health Science Center-HoustonHouston, Texas; 4 Library Director, School of Public Health, University of Texas Health Science Center-HoustonHouston, Texas; 5 Department of Pediatrics, Baylor College of MedicineHouston, Texas

**Keywords:** Human papillomavirus, hybrid capture II, meta-analysis, polymerase chain reaction, test accuracy

## Abstract

The effectiveness of screening programs for cervical cancer has benefited from the inclusion of Human papillomavirus (HPV) DNA assays; which assay to choose, however, is not clear based on previous reviews. Our review addressed test accuracy of Hybrid Capture II (HCII) and polymerase chain reaction (PCR) assays based on studies with stronger designs and with more clinically relevant outcomes. We searched OvidMedline, PubMed, and the Cochrane Library for English language studies comparing both tests, published 1985–2012, with cervical dysplasia defined by the Bethesda classification. Meta-analysis provided pooled sensitivity, specificity, and 95% confidence intervals (CIs); meta-regression identified sources of heterogeneity. From 29 reports, we found that the pooled sensitivity and specificity to detect high-grade squamous intraepithelial lesion (HSIL) was higher for HCII than PCR (0.89 [CI: 0.89–0.90] and 0.85 [CI: 0.84–0.86] vs. 0.73 [CI: 0.73–0.74] and 0.62 [CI: 0.62–0.64]). Both assays had higher accuracy to detect cervical dysplasia in Europe than in Asia-Pacific or North America (diagnostic odd ratio – dOR = 4.08 [CI: 1.39–11.91] and 4.56 [CI: 1.86–11.17] for HCII vs. 2.66 [CI: 1.16–6.53] and 3.78 [CI: 1.50–9.51] for PCR) and accuracy to detect HSIL than atypical squamous cells of undetermined significance (ASCUS)/ low-grade squamous intraepithelial lesion (LSIL) (HCII-dOR = 9.04 [CI: 4.12–19.86] and PCR-dOR = 5.60 [CI: 2.87–10.94]). For HCII, using histology as a gold standard results in higher accuracy than using cytology (dOR = 2.87 [CI: 1.31–6.29]). Based on higher test accuracy, our results support the use of HCII in cervical cancer screening programs. The role of HPV type distribution should be explored to determine the worldwide comparability of HPV test accuracy.

## Introduction

Cervical cancer is a significant cause of morbidity and mortality among women worldwide [1]. Human papillomavirus (HPV) infection is one of the most common sexually transmitted diseases in the world, and infection with high-risk oncogenic types of HPV has been recognized as a necessary cause of cervical cancer and its precursor lesion, cervical intraepithelial neoplasia (CIN) [2, 3]. Fortunately, preventing cervical cancer is possible due to its distinct premalignant stage, and as the introduction of population-based screening programs, cervical cancer incidence and mortality have greatly decreased in developed countries [4–6]. Screening has largely relied on cytology-based tests; however, given their subjective nature as well as low sensitivity and specificity, adding HPV DNA testing to screening programs to improve their efficacy has been proposed [7–9]. Furthermore, the positive predictive value (PPV) of current screening tests is projected to decrease in populations vaccinated against HPV, but this drop in test performance could be mitigated by adding HPV DNA testing to the screening paradigm [10]. There are several ways in which HPV DNA testing might be implemented. First, the HPV DNA assay may be used, either in combination with cytology or alone, as the primary screening method. Studies have shown that HPV testing has a higher sensitivity than cytology, indicating that a longer interval between screenings is possible when including HPV DNA testing in a screening program [11–14]. Second, HPV DNA detection may be used to triage women with cytological abnormalities to determine whether referral for colposcopy is warranted [15, 16]. Lastly, HPV DNA testing may be used as a follow-up to detect residual disease or predict recurrence among women who have been treated for high-grade CIN [17].

The two most common methods used for HPV DNA detection are the Hybrid Capture II (HCII, Qiagen Gaithersburg, Inc., MD) and polymerase chain reaction (PCR) assays. The HCII assay is commercially available and approved for clinical use, and several types of PCR assays have been primarily used in the research setting [18]. Furthermore, both assays have shown high sensitivity to detect high-risk HPV infections but only moderate specificity [18]. Meijer et al. [19] have recommended that any HPV DNA test should have an optimal balance between clinical sensitivity and specificity. Stoler et al. [20] proposed a minimum sensitivity of 92% and a specificity of 85% for any new HPV DNA test. The selection of a screening test is important to detect clinically relevant cases of HPV infection while avoiding the unnecessary cost, stress, and compromise of the cervix to patients associated with overtreating mild cytological abnormalities. Therefore, the goal of this meta-analysis was to compare the clinical performance of HCII and PCR assays in both the screening and diagnostic settings.

## Methods

### Search strategy

Between June 2011 and June 2012, an experienced librarian (H. M. V.) and two investigators (H. N. L. and K. R. D.) conducted a systematic search to identify published studies from 1985 to June 2012. Three main biomedical databases (OvidMedline, PubMed, and Cochrane Library) were searched using the following search terms: ([PCR] OR [HCII] OR [Molecular diagnostic techniques]) AND ([Infections] OR [Papillomavirus infections] OR [Uterine cervical neoplasm] OR [cancer cervix] OR [cervical cancer or cervical neoplasm] OR [HPV or papilloma] OR [HSIL] OR [LSIL] OR [[high grade] OR [low grade]] OR [CIN]).

### Study screening and selection

Inclusion criteria for the meta-analysis were English language reports of studies comparing the sensitivity and specificity of PCR (i.e., MY/PGMY 09/11 or GP5+/6+ or Amplicor) and HCII using either cytologic or histologic results as the gold standard for testing comparison (i.e., Bethesda classification system) in either a screening or follow-up/diagnostic setting. The three specific PCR tests mentioned above were chosen because they are currently the most-used tests. All citations were independently reviewed by two investigators (H. N. L. and K. R. D.). When necessary, authors of a selected article [21] were contacted to obtain further information. Normally, a standard threshold of 1 relative light unit (RLU) or 1 pg/mL of HCII was used to detect the positive presence of HPV DNA. However, to maximize power in our study, we did not restrict by this cutoff.

### Data abstraction and coding

All eligible studies were abstracted independently by two reviewers (H. N. L. and K. R. D.) using a coding system based on the Standards for Reporting Diagnostic Accuracy (STARD) and MOOSE for meta-analysis of observational studies in epidemiology [22, 23]. Any discrepancies were resolved by discussion and consensus between the two investigators. Variables used to present our analysis were grouped into two components, as follows.

#### Characteristics of study participants

Year study conducted, study location, study settings/population/inclusion–exclusion criteria, sample size, study design (i.e., cross-sectional, case–control, cohort, randomized controlled trial), age (i.e., mean, median, range), and race/ethnicity.

#### Testing methods

Gold standard tests (cytology or histology); sample preparation (cervical collection procedures, including collection devices, and DNA preparation for HPV testing methods); types of testing methods (PCR and HCII); test results (HPV prevalence, sensitivity, specificity, [PPV], negative predictive value [NPV], agreement and level of reproducibility [kappa -κ], and their respective 95% confidence interval, CI); and blinding and/or quality control methods.

### Clinical outcomes

Three clinical outcomes were examined, atypical squamous cells of undetermined significance (ASCUS), low-grade squamous intraepithelial lesion (LSIL), which includes HPV infection or mild dysplasia (CIN1), and high-grade squamous intraepithelial lesion (HSIL), which includes moderate (CIN2) and severe dysplasia (CIN3) [24]. Because there were only 10 studies on ASCUS, we merged ASCUS and LSIL to improve the power of analysis.

### Statistical analysis

In this meta-analysis, a study unit was defined as a study having complete information to compare the testing accuracy between PCR and HCII. Depending on the specific PCR test, setting, gold standard, age group, or sample collection method, one article could contribute more than one study unit. For example, an article by Riethmuller et al. [25] compared PCR MY09/11 with HCII in two clinical outcomes (i.e., LSIL and HSIL) and thus generated two study units. Likewise, an article by Stevens et al. [26] contributed eight study units for our analysis because the original analysis included both cytology and histology as gold standards with two clinical outcomes (LSIL vs. HSIL) and two types of PCR (PGMY09/11 and Amplicor).

Complete information from each study was extracted to construct two-by-two tables, which included true-positive, false-positive, true-negative, and false-negative values. The sensitivity was calculated as (true positive)/([true positive]+[false negative]) and specificity was calculated as (true negative)/([true negative]+[false positive]). Forest plots were generated to present, by type of clinical outcome, individual and pooled sensitivity and specificity of each test and to show heterogeneity across studies [27, 28]. Additionally, heterogeneity across studies was examined using Cochran's *Q*-test and the chi-square test [27, 29]. To examine the threshold effect or the difference derived from the use of different cutoffs or thresholds, we computed the Spearman correlation coefficient. This coefficient can be defined as the result of the logit of sensitivity divided by the logit of (1-specificity) [30].

Stratified meta-analysis and meta-regression were used to examine the influence of study characteristics and the magnitude of interstudy heterogeneity on sensitivity and specificity for both PCR and HCII. Stratified meta-analyses were performed for the two clinical outcomes (ASCUS/LSIL and HSIL) by setting (screening vs. follow-up/diagnostic) and by PCR testing technique (i.e., MY/PGMY 09/11, GP5+/6+, and Amplicor). Age is an important variable, particularly age cutoff of 30 years old; however, in our analysis, only two articles reported age using this cutoff (Luu, H. N., K. Adler-Storthz, L. M. Dillon, M. Follen, and M. E. Scheurer, submitted) [31, 32]. We, therefore, decided not to include this variable in the analysis because of low power and inability to generate pooled sensitivity and/or specificity or perform meta-regression [30]. For meta-regression, a generalized linear model was fitted to the data and weighted by the inverse of the variance using Moses and Littenberg methods [32]. Additionally, a random effects model was used to pool variation between studies [33] in the current meta-regression model. For both PCR and HCII, four variables (setting, gold standard, type of lesion, and study location) were included in the meta-regression models. For PCR, an additional variable, type of PCR (i.e., MY/PGMY 09/11, GP5+/6+, Amplicor), was added to the meta-regression model. Diagnostic odds ratios (dOR) and their respective 95% CIs were calculated to determine diagnostic test performance as well as the influence of covariates on test accuracy. The dOR is a measure of the effectiveness of a diagnostic test. It is defined as the ratio of the odds of the test being positive if the subject has a disease relative to the odds of the test being positive if the subject does not have the disease. The dOR was calculated as (sensitivity odds)/(odds of [1-specificity]) [34]. Meta-DiSc [30], a comprehensive software program to evaluate diagnostic and screening tests through meta-analysis, was used to perform the statistical analysis for this study. All statistical tests were two sided and were considered significant at the level of *P* *<* 0.05.

## Results

### Search results

**Figure 1:**
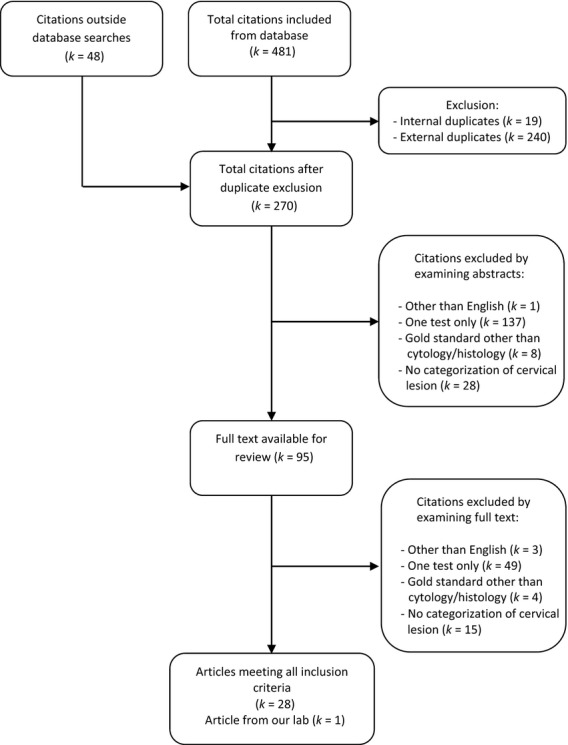
Screening and eligibility evaluation process.

We identified 481 citations from search databases, of which 259 citations were duplicates (Fig. 1). By examining reference lists of those studies, we found an additional 48 citations. We then excluded 175 citations by applying the inclusion criteria to the titles and abstracts and retrieved 95 full-text articles for further review. The review process yielded 28 articles that met all inclusion criteria [14, 21, 25, 26, 31, 35–57]. In addition, we added one manuscript from our own group (Luu et al., submitted), which is currently under review (Fig. 1).

### Characteristics of included articles

A total of 22,417 women were described, with four articles [38, 41, 48, 54] contributing fewer than 100 participants and 10 articles [14, 21, 25, 26, 31, 37, 39, 42, 52, 53] (Luu et al., submitted), more than 500 participants. The article by Schiffman et al. [42] had the largest sample size, with more than 3400 participants.

Over half of the articles (*k* = 16) [14, 21, 25, 31, 35, 36, 39, 43, 45–51, 54] reported results from Europe, with the remainder from Asia-Pacific (*k* = 5) [26, 44, 52, 56, 57], North America (*k* = 6) (Luu et al., submitted) [37, 38, 42, 53, 55], and South America (*k* = 2) [41, 42]. Just under half (*k* = 14) [25, 38–41, 44–46, 49–53] reported studies were conducted in screening settings; 10 were in a follow-up/diagnostic setting [21, 26, 31, 35, 42, 47, 48, 54–56] and the remainder (*k* = 5) (Luu et al., submitted) [14, 36, 37, 43] were in both screening and follow-up/diagnostic settings. Most (*k* = 23) used a cross-sectional study design [21, 25, 26, 31, 35, 36, 38–41, 44–54, 56, 57]; four, a cohort study design (Luu et al., submitted) [14, 37, 43] and two, a randomized controlled trial design [42, 55]. The age range was 15 to 81 years. Half (*k* = 15) [25, 35–40, 43, 44, 46–48, 51, 54, 55] used cytology as the gold standard, 12 (Luu et al., submitted) [14, 21, 31, 42, 45, 49, 50, 52, 53, 56, 57] used histology as the gold standard, and two [26, 41] used both.

The 29 included articles contained 82 PCR study units and 79 HCII study units (Table 1). The uneven number of study units between PCR and HCII resulted from two articles [26, 31] that contained studies comparing more than one type of PCR with HCII.

**Table 1:** Characteristics of included articles

					# Study units and reasons	
					
1st author, date, Design	Year study conducted Location	Study setting Inclusion–exclusion criteria Age	Gold standard test Sample preparation Blinded/Quality control	HPV test HPV prevalence	PCR	HCII
Riethmuller (1999) X-sectional	Aug 1997–May 1998 Besancon & Belfort, France	Screening (*n* = 596) Inclusion–exclusion criteria: not mentioned Age (mean ± SD) Screening group: 36.2 ± 0.6 Colposcopic group: 35.1 ± 0.8	Cytology Sample preparation: • Cervical cells: HPV testing specimens-Digene cervical brush and Digene STM • DNA extraction: phenol-chloroform method. Blinded/Quality Control: Yes	PCR MY09/11 versus HCII HPV prevalence PCR: 37.8%; HC II: 32.9%	2	2
					Clinical outcomes: LSIL versus HSIL	
Bergeron. (2000) X-sectional	Mar 1996–Aug 1998 France	Diagnostic (*n* = 378) Inclusion–Exclusion criteria: not mentioned Age Mean ± SD: 35 ± 10	Cytology Sample preparation: • Cervical cells: ○ Cytologic specimens: wooden spatulas (ectocervices) and cytobrushes (endocervices) ○ HPV testing specimens: cone brush and placed in Eagles' medium (for PCR) or in STM (for HCII) • DNA extraction: low stringency Southern blot hybridization Blinded/Quality Control: Yes	PCR MY09/11 versus HCII HPV prevalence PCR MY09/11: 62.5% HCII: 60.9%	1	1
Venturoli (2002) X-sectional	Year Started: not mentioned Bologna, Italy	Screening & Diagnostic (*n* = 317) Inclusion Criteria + Referred for HPV testing + Tested during a cytological and virological follow-up after conization or hysterectomy for a previous CIN or cervical CA Age (Mean−Median): 38.9−38;	Cytology: Sample preparation: • Cervical cells: ○ PCR testing specimens: Dacron-tipped swab and suspended in PBS ○ HCII: Cytobrush using STM • DNA extraction: Dot-blot hybridization Blinded/Quality Control: Yes	PCR MY09/11 versus HCII HPV Prevalence: Not mentioned	3	3
					Clinical outcomes: ASCUS, LSIL, HSIL	
Yarkin. (2002) X-sectional	Feb 1998-Feb 1999 New York, U.S.A.	Screening (*n* = 94) Inclusion–Exclusion criteria: not mentioned Age Range: 15–51	Cytology: Sample preparation: • Cervical cells: ○ Cytologic specimens exfoliated cervical cells): Ayre spatula and cytobrush ○ HPV testing specimens: 10 mL sterile saline cervicovaginal lavage. • DNA extraction: Phenol-chloroform method Blinded/Quality Control: Yes	PCR MY09/11 versus HCII HPV Prevalence PCR MY09/11: 59.5 (HR) & 79.1% (LR) HC II: 66.4%	3	3
					Settings: screening, Follow-up (0–1 year), follow-up (0–3 year)	
Castle. (2003) Cohort	Apr 1999–Nov 1990 Portland, Oregon, U.S.A.	Screening & Diagnostic (*n* = 1247) Inclusion Criteria: + Women residing in Portland during those time; + Age≥16 Exclusion: + Refusion; + Had hysterectomy + Inadequate specimen for HPV testing; + Had unsatisfactory or missing enrollment cervical smears + Underwent colposcopy rather than Pap smear screening at enrollment Age Mean: 35.9	Cytology Sample preparation: • Cervical cells:Cytologic specimens -PreservCyt solution • DNA extraction: dot-blot hybridization Blinded/Quality Control: not mentioned	PCR MY09/11 versus HCII HPV Prevalence PCR MY09/11: 47.96% HCII (HR HPV): 88.6%	3	3
					Clinical outcomes: ASCUS, LSIL, HSIL	
Kulmala (2004) X-sectional	1998 Moscow, Novgorod (Russia), Minsk (Belarus), and Riga (Latvia)	Screening (*n* = 1511) Inclusion Criteria + Women participated in cervical cancer screening + Women attended gynecology outpatient clinics with different indications + Women examined at STD clinics Age (Mean ± SD): 32.9 ± 11.0	Cytology Sample preparation • Cervical cells: HPV testing – sampling kit for HCII assay • DNA extraction: High-salt method Blinded/Quality Control: Yes	PCR GP05+/06+ versus HCII HPV Prevalence (High-risk HPV) PCR: 36.6%; HCII: 33.7%	1	1
Nonogaki (2004) X-sectional	2002–2004 São Paulo, Brazil	Screening (*n* = 261) Inclusion Criteria Women who had no history of HPV infection or any other cervical pathology Age (Mean ± SD): 36.99 ± 9.88	Cytology Sample preparation • Cervical cells ○ Cytologic specimens: Scored cervical brush ○ HPV testing specimens: DNA Citoligh System • DNA extraction: GFX Genomic Blood DNA Purification Kit (Amersham Pharmacia Biotech Inc., Piscataway, NJ, U.S.A.); eluted to Tris-HCl (100 μL) & EDTA (500 mmol/L) Blinded/Quality Control: Yes	PCR PGMY09/11 versus HCII HPV Prevalence PCR: 51.92%; HCII: 49.42%	3	3
					Clinical outcomes: ASCUS, LSIL, HSIL	
Nonogaki (2005) X-sectional	2002–2004 São Paulo, Brazil	Screening (*n* = 45) Inclusion Criteria No history of HPV infection or any other cervical pathology Age Mean: 37.0	Cytology/Histology Sample preparation • Cervical cells: ○ Cytologic specimens: Scored cervical brush ○ HPV testing specimens: DNA Citoligh System • DNA extraction: phenol-chroroform method Blinded/Quality Control: Yes	PCR GP5+/6+ versus HCII HPV Prevalence PCR: 20.0%; HCII: 28.9%	5	5
					Clinical outcomes: ASCUS, LSIL, HSIL Gold standard: Cytology versus Histology	
Schiffman (2005) Randomized trial	November 1996–December 1998 Pittsburgh, Oklahoma, Seattle, U.S.A.	Diagnostic (*n* = 5060; 3488- ASCUS & 1572- LSILs) Inclusion Criteria + Had a cytologic diagnosis of ASCUS or LSIL within 6 months of enrollment; +≥18 years of age; + No prior hysterectomy; + No known history of ablative or excisional therapy to the cervix; + Not pregnant, and + Able to provide informed consent and likely to participate for the full duration of the trial Age (Mean – Median) ASCUS: 28.8−26 LSIL: 24.8−23	Histology Sample preparation • Cervical cells: ○ Cytologic specimens: Papette broom and PreserCyt ○ HPV testing specimens: Dacron swab with Digene STM • DNA extraction: phenol-chroroform method Blinded/Quality Control: Yes	PCR PGMY09/11 versus HCII HPV Prevalence PCR: 58.2% (any type); 53.2% (multiple types) HC II: 50.6% (ASCUS); 79.9% (LSIL)	5	5
					Settings: Screening, 6, 12, 18, 24 month follow-up	
Söderlund-Strand (2005) Cohort	Year started: not mentioned, Västmanland county, Sweden	Screening & Diagnostic (*n* = 171) Inclusion Criteria Attended population-based cervical cancer screening, referred because of atypical smears. Age: not mentioned	Cytology Sample preparation • Cervical cells: ○ Cytologic specimens: Cytobrush ○ HPV testing specimens: Cytobrush and Digene kit with STM • DNA extraction: Freeze–thaw method, using Tris-HCl (10 mmol/L, pH 7.4) for resuspension. Blinded/Quality Control: not mentioned	PCR GP5+/6+ versus HCII HPV Prevalence Not mentioned	4	4
					Clinical outcomes: LSIL, HSIL Settings: Screening versus follow-up	
Huang (2006), X-sectional	January 2001–September 2004 Taiwan	Screening (*n* = 354) Inclusion Criteria Women attended Department of Gynecology & Obstetrics, Changung Memorial Hospital. Age: not mentioned	Cytology Sample preparation • Cervical cells: ○ Cytologic specimens: Swab ○ HPV testing specimens: Digene kit using STM • DNA extraction: Samples lysed by adding proteinase K (20 μL), AL buffer (0.2 mL), and incubated at 700°C for 10 min. Ethanol 96–100% (0.2 mL) added for precipitation. Sample mixture was loaded onto DNeasey Mini Spin column. DNA solution (100 μL) was eluted & 1 μL aliquot was used. Blinded/Quality Control: Yes	PCR GP5+/6+ versus HCII HPV Prevalence HCII: 33.9%; PCR GP5+/GP6+: 38.7%	2	2
					Clinical outcomes: LSIL, HSIL	
Carozzi (2007) X-sectional	Yeart started: not mentioned Florence, Italy	Screening (*n* = 936) Inclusion Criteria Women aged 25–64 Age Range: 25–64	Histology Sample preparation • Cervical cells: HPV testing specimens – Digene kit with PreservCyt medium • DNA extraction: Manufacturer's instructions for Roche Amplicor test. Blinded/Quality Control: not mentioned	PCR Amplicor (Roche) versus HCII HPV Prevalence PCR (Amplicor-Roche): 39.3%; HCII: 45.6%	1	1
Fontaine (2007) X-sectional	January 2002–December 2004 Charleroi, Belgium	Screening (*n* = 162) Inclusion Criteria Women attended Department of Gynecology, CHU de Charleroi (Charleroi, Belgium). Age (Mean ± SD): 37.3 ± 10.8	Cytology Sample preparation • Cervical cells: ○ Cytologic specimens: Cervexbrush HPV testing specimens: Digene kit with PBS and suspended in Easyfix solution. • DNA extraction: Freeze–thaw method, using Tris-HCl (10 mmol/L, pH 7.5) for resuspension. Blinded/Quality Control: Yes	PCR PGMY09/11 versus HCII HPV Prevalence Not mentioned	3	3
					Clinical outcomes: ASCUS, LSIL, HSIL	
Halfon (2007) X-sectional	2000 5 centers (Marseille, Aix les Bains, Lyon, Le Havre, and Meylan), France	Diagnostic (*n* = 271) Inclusion Criteria Patients with ASCUS Age: not mentioned	Cytology Sample preparation • Cervical cells: oCytologic specimens: LBC ○ HPV testing specimens: ThinPrep using PreservCyt LBC medium. • DNA extraction: AmpliLute liquid medium extraction kit was used. Blinded/Quality Control: not mentioned	PCR Amplicor (Roche) versus HCII HPV Prevalence PCR (Amplicor-Roche): 58% HC II: 59%	2	2
					HR and intermediate risk HPV types	
Stenvall (2007) X-sectional	September 2004–May 2005 Årsta and Svartbäcken, Uppsala, Sweden	Diagnostic (*n* = 43) Inclusion Criteria Previous abnormal cytology Age (Mean): 35.8	Cytology Sample preparation • Cervical cells: ○ Cytologic specimens: Self-sampling device and cytobrush. ○ HPV testing specimens: Digene test kit. • DNA extraction: Specimens were dissolved in 150 μL of digestion buffer containing Tris (50 nmol/L), EDTA (1 mmol/L), & Tween-20. Distilled water & Proteinase K were added. Blinded/Quality Control: not mentioned	PCR GP5+/6+ versus HCII HPV Prevalence PCR: 40% HCII: 37%	2	2
					Clinical outcomes: ASCUS, HSIL	
Stevens (2007) X-sectional	May 2001–June 2005 Melbourne, Australia	Diagnostic (*n* = 1679) Inclusion Criteria + Anormal Pap smears or postcoital bleeding; + Abnormal-looking cervix, + Ongoing review of previous abnormality Age (Mean ± SD): 29.8 ± 7.9	Cytology and Histology Sample preparation • Cervical cells: ○ Cytologic specimens: Cervex brush and rinsed into ThinPrep vials containing PreservCyt fixative solution. • DNA extraction: Automated MagNA PureLC (MP) isolation and purification system (Roche Molecular System) with modified protocol. Blinded/Quality Control: not mentioned	PCR Amplicor (Roche) versus HCII HPV Prevalence Amplicor (Roche): 73.3% HC II: 64%	8	4
					PCR: Clinical outcomes: LSIL, HSIL Gold standard: Cytology, histology HCII: Only available for Cytology (HSIL) and Histology (LSIL vs. HSIL)	
Cuzick (2008) Cohort	April 1994–September 1997 U.K.	Screening (*n* = 2981) Inclusion Criteria Regular screening recruited through general practitioners Age: ≥35	Histology Sample preparation • Cervical cells: ○ Cytologic specimens: Aylesbury brush. ○ HPV testing specimens: Digene with PBS (for PCR) and STM (for HCII). • DNA extraction: not mentioned Blinded/Quality Control: Yes	PCR MY09/11 versus HCII (using cutoff 1 pg/mL, 2 pg/mL, 4 pg/mL) HPV Prevalence Not mentioned	2	6
					Settings: Screening versus Diagnostic HCII: standard cutoff 1 pg/mL, 2 pg/mL, 4 pg/mL	
De Francesco (2008)	May 2005–May 2006 Spedali Civili, Italy	Screening (*n* = 213) Inclusion criteria: • Not currently pregnant; ≥ 2 months postpartum • Intact uterus and no current referral for hysterectomy • Never been treated for squamous intraepithelial lesions • No history of chronic diseases (e.g., renal failure, diabetes, cancer or gastrointestinal malabsorption) Age (Median): 35.6	Histology Sampling preparation • Cervical cells: ○ Cytologic specimens: Cytobrush. ○ HPV testing specimens: Digene kit using STM. • DNA extraction: not mentioned Blinded/Quality Control: Yes	PCR Amplicor (Roche) versus HCII HPV Prevalence *Overall:* PCR (Amplicor): 75.2% HCII: 73.3% *ASCUS:* PCR (Amplicor): 67.2% HC II: 64% *LSIL:* PCR (Amplicor): 74.1% HC II: 69.8%	2	2
					Clinical outcomes: LSIL versus HSIL	
Klug (2008) Danish study: cohort German study: X-sectional	May 1991–January 1995 (Danish younger study) Oct 1993–Jan 1995 (Danish older study) (Copenhagen, Denmark) Dec 1998–Dec 2000 Hanover, Tuebingen (Germany) Study	Screening (*n* = 324) Inclusion criteria *Danish study* + Normal cytology at baseline; + ≥1 smear during the follow-up period Exclusion criteria: + Participated through telephone interview; + Inadequate or missing baseline smear; + Abnormal smear at baseline; + Being followed for an abnormal Pap smear diagnosed within 1 year before baseline + Did not contribute a cervical sample at baseline *German study* + Attended for routine annual cervical cancer screening; + ≥30 years olds; + Not undergone a hysterectomy; + No history of atypical cytology, CIN, or treatment for cervical disease in the preceding year; + Not pregnant Exclusion criteria: + Genital warts; + History of conization or hysterectomy; + Pregnant (*n* = 11); + Abnormal cytology within 1 year of study entry + <30 years old; + Not given written consent Age: *Danish study* (Median): HR HPV (−): 45.2; HR HPV (+): 44.4 *German study*: ≥30 years old	Histology Sample preparation • Cervical cells: ○ Cytologic specimens: Cotton-tipped swab. ○ HPV testing specimens: Digene kit using STM. • DNA extraction: phenol-chloroform method. Blinded/Quality Control: not mentioned	PCR PGMY09/11 & PCR GP5+/6+ versus HCII HPV Prevalance PCR PGMY09/11: 72.1% PCR GP5+/6+: 62.6% HCII: 80.2%	2	2
					2 types of PCR: PGMY09/11 & GP5+/6+	
Mo (2008) X-sectional	Year started: not mentioned France	Screening (*n* = 471) Inclusion Criteria Women attending routine cytological screening program. Age (Median): 34	Cytology Sample preparation • Cervical cells: Samples were collected using PreservCyt solution • DNA extraction: Following manufacturer's instructions from Roche Amplicor HPV test DNA extraction. Blinded/Quality Control: Yes	PCR Amplicor (Roche) versus HCII HPV Prevalence PCR Amplicor (Roche): 53% HCII: 53%	3	3
					Clinical outcomes: ASCUS, LSIL, and HSIL	
Szareswki (2008) X-sectional	August 2005–January 2007 London, U.K.	Diagnostic (*n* = 953) Inclusion Criteria + ≥1 abnormal cervical smears; + Not pregnant + Not been treated previously for CIN, nor had hysterectomy. Age (Median): 29.9	Histology Sample preparation • Cervical cells: Cervex broom and placed in PreservCyt transport medium. • DNA extraction: not mentioned Blinded/Quality Control: Yes	PCR MY09/11 & PCR Amplicor (Roche) versus HCII HPV Prevalence Not mentioned	2	1
					2 types of PCR: MY09/11 & Amplicor	
Bhatla (2009) X-sectional	January 2003–June 2006 New Delhi, India	Screening (*n* = 512) Inclusion Criteria + Women complaints of vaginal discharge or irregular bleeding; + Women with unhealthy cervix on exam. Age (Median): 36	Histology Sample preparation • Cervical cells: ○ Cytologic specimens: Self-sampling device and cytobrush. + Patient self-collected: Brush + Physician collected: Ayre spatula and brush ○ HPV testing specimens: Digene test kit with STM. • DNA extraction: Specimens were dissolved in 150 μL of digestion buffer containing Tris (20 nmol/L), EDTA (1 mmol/L), Tween-20 10%(100 μL), & Proteinase K 20 mg/ mL (200 μL at 65°C for 1, heat inactivation at 95°C for 10 min). DNA was precipitated with ethanol & ammonium acetate at −20°C overnight. Blinded/Quality Control: not mentioned	PCR PGMY09/11 versus HCII HPV Prevalence 18.75% any HPV; 14.3% HR-HPV	4	4
					Clinical outcomes: LSIL versus HSIL Sample collection types: Physician collected versus Patient self-collected	
Feng (2009) Cohort Study 1 X-sectional Study 2	December 2000–June 2005 (Study 1) December 1997–October 2000 (Study 2) Washington, U.S.A.	Screening (*n* = 267) Inclusion Criteria *Study 1:* Female University of Washington undergraduates + Age 18–22; + Never had vaginal intercourse or had first intercourse with 1 male partner within the previous 3 months; + Had a cervix; + Not pregnant; + In good health; + Be able to provide written consent *Study 2*: + Age 18–50; + No history of hysterectomy; + No history of chronic immune suppression; + No history of treatment for cervical neoplasia; + Agreed to provide written consent Age (Mean ± SD) 21.9 ± 1.7 (Study 1); 24.1 ± 5.9 (Study 2)	Histology Sample preparation • Cervical cells: ○ Cytologic specimens: Dacron-tipped swab (Study 1); Cytobrush (Study 2). ○ HPV testing specimens: Swabs kit with STM (Both studies). • DNA extraction: Qiagen QIAquick PCR purification column and QIAamp column were used. Blinded/Quality Control: not mentioned	PCR PGMY09/11 versus HCII HPV Prevalence Not mentioned	2	2
					Clinical outcomes: LSIL versus HSIL	
Weynand (2009) X-sectional	Year started: not mentioned Belgium	Diagnostic *n* = 47) Inclusion Criteria Women underwent conization. Age (Mean): 40	Histology Sample preparation • Cervical cells: ○ Cytologic specimens: Cervex broom and placed in PreservCyt transport medium. • DNA extraction: Using QIAMP DNA mini Kit (Qiagen, Valencia, CA, U.S.A.). Blinded/Quality Control: not mentioned	PCR GP5+/GP6+ versus HCII HPV Prevalence 74.9% (any HR HPV type)	1	1
Cuzick (2010) X-sectional	August 2005–January 2007 London, U.K.	Diagnostic (*n* = 858) Inclusion Criteria + ≥1 abnormal cervical smears; + Not pregnant • Not been treated previously for CIN, nor had hysterectomy Age: not mentioned	Cytology Sample preparation • Cervical cells: ○ Cytologic specimens: Cervex brush ○ HPV testing specimens: Digene test kit with STM. • DNA extraction: not mentioned. Blinded/Quality Control: not mentioned	PCR GP5+/GP6+ versus HCII HPV Prevalence Not mentioned	6	6
					Clinical outcomes: ASCUS, LSIL, HSIL Medium: PBS and STM	
Castle (2011) Randomized control trial	1997–2001 Multisite, U.S.A.	Diagnostic (*n* = 473) Inclusion Criteria Referred for ASCUS or LSIL conventional cytology. Age (Median): 25	Cytology Sample preparation • Cervical cells: ○ Cytologic specimens: PresevCyt. ○ HPV testing specimens: Digene test kit with STM. • DNA extraction: BD Viper XTR ferric oxide (FOX) particle DNA binding and magnetic extraction. Blinded/Quality Control: Yes	PCR PGMY09/11 versus HCII HPV Prevalence PCR: 50.4%	1	1
Lin (2011) X-sectional	August 1999–March 2004 Multicenter, Taiwan	Diagnostic (*n* = 220) Inclusion Criteria Abnormal cervical cytology. Age (Mean ± SD): 45.66 ± 0.68	Histology Sample preparation • Cervical cells: ○ Cytologic specimens: Cervical scrape, Accelon Combi broom. ○ HPV testing specimens: Scrapes with STM. • DNA extraction: Reverse line blot extraction. Blinded/Quality Control: Yes	PCR PGMY09/11 versus HCII HPV Prevalence Not mentioned	2	2
					Clinical outcomes: LSIL versus HSIL	
Wong (2011) X-sectional	Baseline: March 1, 1998–February 28, 2000 Follow-up: March 1, 2000–February 28, 2002 Hongkong, China	Screening (*n* = 82) Inclusion Criteria + Either underwent colposcopic history findings or repeated cytology + Or underwent both colposcopic and cytology findings. Age (Mean): 35.75	Histology Sample preparation • Cervical cells: ○ Cytologic specimens: Papette broom, then rinsed into ThinPrep. ○ HPV testing specimens: Abbott mSample Preparation kit (for Real Time PCR) and Digene kit using STM (for HCII). • DNA extraction: Abbott mSample Preparation System_*DNA*_ where sample is lysed with chaotropic reagents and DNA is captured with magnetic microparticle technology. Blinded/Quality Control: not mentioned	PCR GP5+/GP6+ versus HCII HPV Prevalence PCR GP5+/6+: 53.2% HC II: 49.6%;	1	1
Luu (Submitted-2013) Cohort	1998–2005 Houston-U.S.A., British Columbia, Canada	Screening & Diagnostic (*n* = 1850) Inclusion Criteria: Aged ≥18 *Screening*: Normal Pap smear and no cervical treatment *Diagnostic*: Recent or past abnormal Pap smear; Had hysterectomy or pregnant at the time of the enrollment Age (Mean ± SD): 44.12 ± 0.38 (Screening); 33.65 ± 0.40 (Diagnostic)	Histology Sample preparation • Cervical cells: ○ Cytologic specimens: Cytobrush. ○ HPV testing specimens: Digene test kit. • DNA extraction: QIAamp DNA Mini Kit (Qiagen, Valencia, CA, U.S.A.). Blinded/Quality Control: Yes	PCR MY09/11 versus HCII HPV Prevalence Screening versus Diagnostic LSIL: HCII (0.8% vs. 11%) PCR (8% vs. 22%) HSIL: HCII (1.2% vs. 26%) PCR (1.2% vs. 26%)	6	6
					Clinical outcomes: ASCUS, LSIL, HSIL Settings: Screening versus Diagnostic	

Sen, sensitivity; Spe, specificity; PPV, positive predictive value; NPV, negative predictive value; STM, special transport medium; PBS, phosphate-buffered saline solution; ASCUS, atypical squamous cell of undetermined significance; LSIL, low-grade squamous cell intraepithelial lesion; HSIL, high-grade squamous cell intraepithelial lesion; CIN, cervical intraepithelial neoplasia; HR HPV, high-risk human papillomavirus; LR HPV, low-risk human papillomavirus.

### Accuracy of the tests

**Figure 2:**
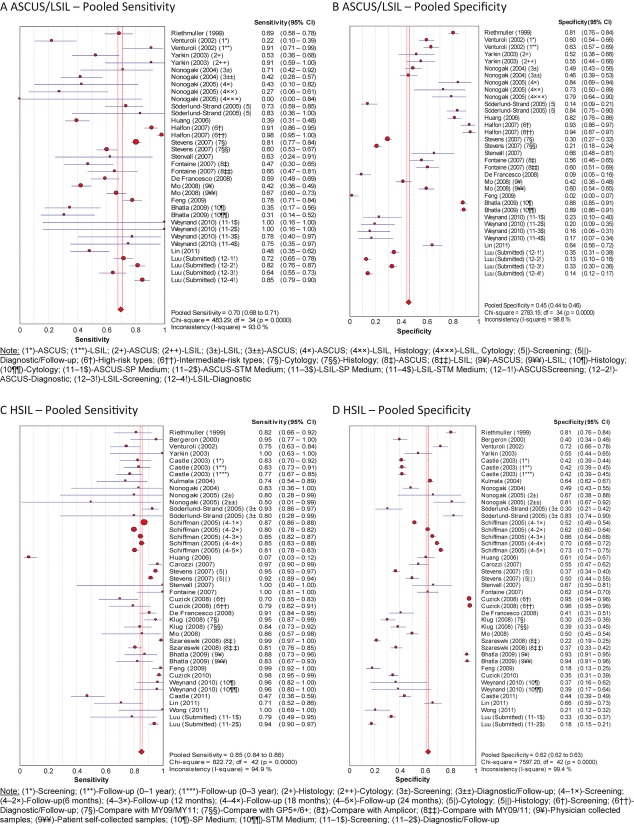
Forest plots of PCR performance in identifying clinical lesions.

**Figure 3:**
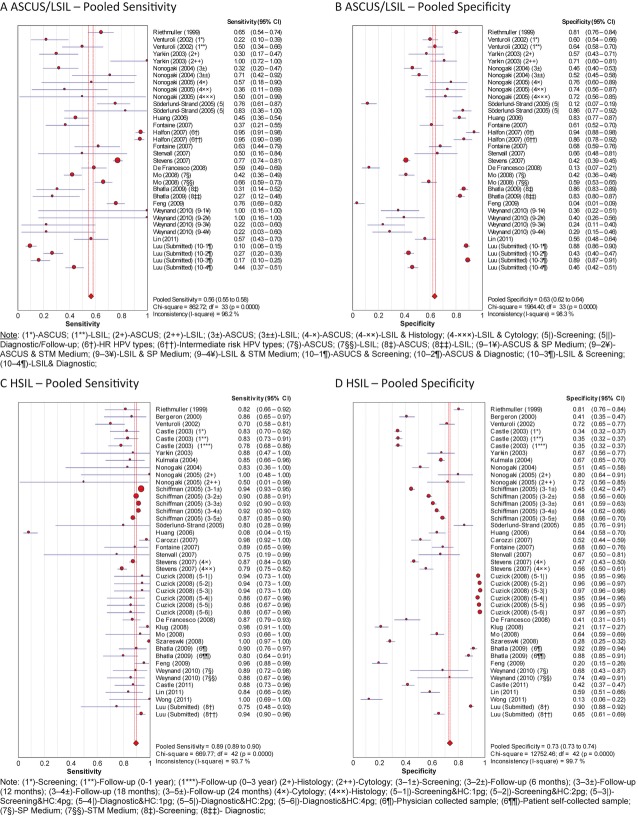
Forest plots of HCII performance in identifying clinical lesions.

In detecting ASCUS/LSIL, PCR was more sensitive (Fig. 2A and B) but less specific than HCII (Fig. 3A and B) (pooled sensitivity: 0.70 [95% CI: 0.68–0.71], pooled specificity: 0.45 [95% CI: 0.44–0.46] for PCR and pooled sensitivity: 0.56 [95% CI: 0.55–0.58], pooled specificity: 0.63 [95% CI: 0.62–0.64] for HCII. PCR GP5+/6+, however, was both less sensitive and less specific than HCII in detecting ASCUS/LSIL lesions (pooled sensitivity: 0.51 [95% CI: 0.45–0.58], pooled specificity: 0.56 [95% CI: 0.53–0.60]) (Table 2). HCII was more sensitive and specific than PCR in detecting HSIL (Fig. 2C and D, Fig. 3C and D) (pooled sensitivity: 0.89 [95% CI: 0.89–0.90] and pooled specificity: 0.73 [95% CI: 0.73–0.74] for HCII vs. pooled sensitivity: 0.85 [95% CI: 0.84–0.86] and pooled specificity: 0.62 [95% CI: 0.62–0.63] for PCR). Consistent with the overall analysis, HCII showed a much higher sensitivity and specificity than PCR GP5+/6+ in detecting HSIL in the screening setting (pooled sensitivity: 0.90 [95% CI: 0.88–0.91] and pooled specificity: 0.77 [95% CI: 0.77–0.78] for HCII vs. pooled sensitivity: 0.55 [95% CI: 0.50–0.60] and pooled specificity: 0.58 [95% CI: 0.56–0.60] for PCR) (Table 2).

**Table 2:** Pooled sensitivity and specificity of PCR and HCII by lesion types (Bethesda classification)

Lesion types	# Study units	Pooled sensitivity (95% CI)	Pooled specificity (95% CI)
PCR			
ASCUS/LSIL	35	0.70 (0.68−0.71)	0.45 (0.44–0.46)
MY/PGMY 09/11	17^1^	0.69 (0.67–0.72)	0.48 (0.47–0.50)
Screening	14	0.64 (0.61–0.67)	0.56 (0.55–0.58)
Diagnostic/Follow-up	5	0.75 (0.72–0.79)	0.33 (0.31–0.35)
GP5+/6+	11	0.51 (0.45–0.58)	0.56 (0.53–0.60)
Screening	5	0.47 (0.40–0.54)	0.62 (0.57–0.66)
Diagnostic/Follow-up	6	0.77 (0.60–0.90)	0.48 (0.42–0.54)
Amplicor	7	0.73 (0.70–0.75)	0.36 (0.34–0.38)
Screening	3	0.55 (0.51–0.60)	0.44 (0.40–0.48)
Diagnostic/Follow-up	4	0.81 (0.79–0.83)	0.33 (0.31–0.36)
HSIL	43	0.85 (0.84–0.86)	0.62 (0.62–0.63)
MY/PGMY 09/11	25	0.84 (0.83–0.85)	0.66 (0.65–0.66)
Screening	13	0.87 (0.86–0.88)	0.67 (0.66–0.68)
Diagnostic/Follow-up	12	0.82 (0.81–0.83)	0.65 (0.64–0.66)
GP5+/6+	12	0.75 (0.72–0.79)	0.54 (0.52–0.55)
Screening	7	0.55 (0.50–0.60)	0.58 (0.56–0.60)
Diagnostic/Follow-up	5	0.97 (0.95–0.99)	0.43 (0.39–0.46)
Amplicor	6	0.94 (0.93–0.95)	0.38 (0.36–0.40)
Screening	3	0.93 (0.88–0.96)	0.50 (0.46–0.53)
Diagnostic/Follow-up	3	0.94 (0.93–0.96)	0.34 (0.32–0.36)
HCII			
ASCUS/LSIL	34	0.56 (0.55–0.58)	0.63 (0.62–0.64)
Screening	22	0.46 (0.43–0.48)	0.71 (0.70–0.72)
Diagnostic/Follow-up	13	0.67 (0.65–0.70)	0.51 (0.49–0.52)
HSIL	43	0.89 (0.89–0.90)	0.73 (0.73–0.74)
Screening	23	0.90 (0.88**–**0.91)	0.77 (0.77**–**0.78)
Diagnostic/Follow-up	21	0.89 (0.88**–**0.90)	0.70 (0.70**–**0.71)

ASCUS, atypical squamous cell of undetermined significance; LSIL, low-grade squamous intraepithelial lesion; HSIL, high-grade squamous intraepithelial lesion.

1 The Venturoli et al. study 2002 contributes 2 study units in the screening group and 2 study units in the diagnostic group.

### Heterogeneity and meta-regression

The Spearman correlation coefficients of HCII and PCR were 0.005 (*P* = 0.96) and 0.234 (*P* = 0.03), respectively. Further analysis showed that besides a threshold effect for PCR, both HCII and PCR had heterogeneity due to various factors (HCII: *χ*^2^ = 5184.26, *P*_heterogeneity_ < 0.0001, *I*^2^ = 98.5% and PCR: *χ*^2^ = 2540.74, *P*_heterogeneity_ < 0.0001, *I*^2^ = 96.8%).

Setting was not a source of heterogeneity for either PCR or HCII accuracy (Table 3). PCR has higher accuracy to detect HSIL than to detect ASCUS/LSIL (dOR: 5.60 [95% CI: 2.87–10.94], *P* < 0.0001). PCR MY/PGMY 09/11 and Amplicor showed 2.75 (95% CI: 1.16–6.53, *P* = 0.02) and 3.01 (95% CI: 1.05–8.63, *P* = 0.04) times higher accuracy in detecting dysplasia lesions than PCR GP5+/6+ (Table 3). Also, PCR has higher accuracy in detecting dysplasia lesions in Europe than in Asia-Pacific Region or North America (dOR: 2.66 [95% CI: 1.16–6.53], *P* = 0.03 and dOR: 3.78 [95% CI: 1.50–9.51], *P* = 0.005 [data not shown], respectively).

**Table 3:** Multivariable meta-regression of PCR and HCII performance

Variable	Coeff.	Standard error	*P-*value	dOR (95% CI)
PCR				
Settings				
Screening	Ref.	Ref.	Ref.	Ref.
Diagnostic/Follow-up	0.62	0.35	0.08	1.87 (0.92**–**3.77)
Gold Standard				
Cytology	Ref.	Ref.	Ref.	Ref.
Histology	−0.03	0.35	0.93	0.97 (0.48–1.96)
Lesion (dOR)				
ASCUS/LSIL	Ref.	Ref.	Ref.	Ref.
HSIL	1.72	0.34	**<0.0001**	**5.60 (2.87–10.94)**
PCR types				
GP5+/6+	Ref.	Ref.	Ref.	Ref.
MY/PGMY 09/11	1.01	0.43	**0.02**	**2.75 (1.16–6.53)**
Amplicor	1.10	0.53	**0.04**	**3.01 (1.05–8.63)**
Location				
Asia–Pacific	Ref.	Ref.	Ref.	Ref.
North America	−0.35	0.50	0.48	0.70 (0.26**–**1.89)
South America	0.42	0.72	0.56	1.53 (0.36**–**6.44)
Europe	0.98	0.45	**0.03**	**2.66 (1.16–6.53)**
HCII				
Settings				
Screening	Ref.	Ref.	Ref.	Ref.
Diagnostic/Follow-up	0.43	0.38	0.25	1.54 (0.73**–**3.25)
Gold Standard				
Cytology	Ref.	Ref.	Ref.	Ref.
Histology	1.05	0.39	**0.009**	**2.87 (1.31–6.29)**
Lesion				
ASCUS/LSIL	Ref.	Ref.	Ref.	Ref.
HSIL	2.02	0.39	**<0.0001**	**9.04 (4.12–19.86)**
Location				
Asia-Pacific	Ref.	Ref.	Ref.	Ref.
North America	−0.11	0.55	0.84	0.89 (0.30**–**2.70)
South America	0.84	0.79	0.29	2.32 (0.48**–**11.22)
Europe	1.40	0.54	**0.01**	**4.08 (1.39–11.91)**

ASCUS, atypical squamous cell of undetermined significance; LSIL, low-grade squamous intraepithelial lesion; HSIL, high-grade squamous intraepithelial lesion; dOR, diagnostic odds ratio.

Bold values are statistically significant.

For HCII accuracy, we did find that the use of gold standard methods was contributed to heterogeneity (dOR = 2.87 [95% CI: 1.31–6.29], *P* = 0.009, comparison between histology and cytology, cytology = reference group). The HCII is 9.04 times (95% CI: 4.12–19.86) more accurate to detect HSIL than ASCUS/LSIL. Similarly, HCII had higher accuracy in detecting lesions in Europe than in Asia-Pacific Region or North America (dOR: 4.08 [95% CI: 1.39–11.91], *P* = 0.01 and dOR: 4.56 [95% CI: 1.86–11.17], *P* = 0.001 [data not shown in Table 3], respectively).

## Discussion

In the current meta-analysis, we examined the pooled sensitivity and specificity of HCII and PCR in the screening and follow-up/diagnostic settings, following the 2001 Bethesda Classification (i.e., ASCUS, LSIL, and HSIL). We identified 28 published articles and our own manuscript that compared HCII and PCR in the same report. We found that in detecting ASCUS/LSIL, HCII was less sensitive but more specific than PCR. We also found that HCII was both more sensitive and more specific in detecting HSIL than was PCR (both in screening and diagnostic settings). Clinical outcome and study location were sources of heterogeneity for the accuracy of both PCR and HCII. Additionally, PCR types and gold standard were sources of interstudy variability of the accuracy of PCR and HCII, respectively.

To our knowledge, this is the first meta-analysis that directly compares the accuracy of HCII and PCR in screening and diagnostic settings and across two clinical outcomes. In 2004, Arbyn and associates [9] conducted a meta-analysis and reported that for ASCUS detection, HCII alone was less sensitive but more specific (pooled sensitivity: 0.95 [95% CI: 0.93–0.97] and pooled specificity: 0.67 [95% CI: 0.58–0.76]) than HPV DNA testing ([all] pooled sensitivity: 0.84 [95% CI: 0.78–0.91] and pooled specificity: 0.73 [95% CI: 0.63–0.83]) by PCR. The major difference between our study and Arbyn's [9] is that they reported the test accuracy of HCII and a combination of HPV DNA tests (i.e., HCII, HCI, and PCR). Therefore, HCII and PCR were not compared directly. The other difference between our studies is that they [9] included test results from studies of a single test, whereas our review was restricted to studies that compared HCII and PCR. Because findings of the test accuracy come from the same population, we sought to minimize the source of interstudy heterogeneity. Furthermore, Arbyn et al. [9] included 17 articles from 1992 to 2002, whereas we identified 29 articles from 1999 to 2011. During the 1999–2011 period, HCII has been used more than it was during the period covered by Arbyn, and our meta-analysis included only one article [35] that was also in Arbyn's meta-analysis [9]. Additionally, the meta-analysis by Arbyn et al. [9] was restricted to cross-sectional studies while ours expanded to other study designs (i.e., cohort and randomized controlled trial). As Sherman et al. [58] recommended, a longitudinal study design helps to detect missed lesions by repeated cytology before invasive cancer occurs. The article by Schiffman et al. [42] (the ASCUS-LSIL Triage Study-ALTS), included in our meta-analysis, reported that during 2 years of follow-up during which study participants were asked to visit at 6-month intervals, HCII showed higher sensitivity than and comparable specificity with PCR. Finally, while Arbyn et al. [9] restricted their analysis to ASCUS, we expanded ours to more important clinical outcomes (i.e., LSIL and HSIL). With the availability of the test accuracy for these two clinical outcomes, misdiagnosis or overtreatment due to screening test results can be avoided.

Type of clinical outcome was a source of interstudy heterogeneity in our meta-analysis. Accordingly, both tests appeared to have higher accuracy to detect HSIL than to detect ASCUS/LSIL. This is expected because of the cytological and histological differences between these lesions, which are driven by HPV. The other source of heterogeneity within HCII studies was the choice of the gold standard (i.e., cytology vs. histology). For example, an article by Stevens et al. [26] reported that when cytology is used as the gold standard, the sensitivity and specificity of HCII was 0.87 (95% CI: 0.86–0.89) and 0.47 (95% CI: 0.44–0.49), respectively. On the other hand, test accuracy is different when histology was used as the gold standard (sensitivity: 0.79 [95% CI: 0.76–0.82] and specificity: 0.56 [95% CI: 0.53–0.59]). Our results showing a higher HCII test accuracy if histology is used as the gold standard supports the findings of Sherman et al. [58] that a lead time bias occurs if repeat cytology is performed, particularly among women with ASCUS or LSIL. Consequently, one might only detect a smaller proportion of CIN3 lesions that do not have sufficient features associated with invasive cancer and miss a larger proportion of lesions usually associated with invasive cancer [58].

Study location is another important source of heterogeneity in our findings. We found higher accuracy of both PCR and HCII tests between European and Asia-Pacific Region studies and between European and North American studies. We thought this might be related to the HPV type distribution in the different study locations. This interpretation is supported by the meta-analysis by Smith and associates [59] showing that although 16 and 18 presence in all regions, there is difference in HPV specific types in different regions, from 16, 31, 33, and 18 in Europe; 16, 58, 18, and 51 in Asia-Pacific Region; 16, 18, 31, and 35 in North America.

Results from several large randomized controlled trials in Europe [60–62], North America [61, 63, 64], and Asia-Pacific [65] supported the use of HPV DNA testing over cytology for cervical cancer screening. For example, the 5-year Population-Based Screening Study Amsterdam [62], which included approximately 45,000 women aged 26–45, reported that HPV cotesting is more sensitive than cytology alone to detect baseline CIN2 and 3 and to detect cervical cancer at the end of the trial. Another randomized trial [65] of approximately 132,000 women, aged 30–59, conducted over 7 years in rural India also reported substantially higher sensitivity of HCII over cytology. Our analysis showed that while PCR was more sensitive but less specific than HCII in detecting ASCUS/LSIL, HCII was more sensitive and more specific than PCR in detecting HSIL. Our findings, therefore, support the use of HCII because of its clinical relevance. The 2006 Consensus Guidelines for the Management of Women with CIN or Adenocarcinoma in situ [66] recommended that patients with CIN1 preceded by ASCUS or LSIL be followed-up with either HPV DNA testing every 12 months or repeated cytology every 6–12 months. CIN1 is heterogeneous in that it may be ASCUS; however, it may also include LSIL, ASC-H, or even HSIL [67]. Both high-risk and low-risk HPV types may be present in CIN1 lesions [68, 69]. Additionally, several studies show that in the absence of treatment there is a high rate of spontaneous regression of low-grade cervical lesions [70–72] and that CIN1 unusually progresses to CIN2 or CIN3 [62, 73]. For example, a study by Moscicki et al. [71] reported that in more than 91% of adolescents and young women with LSIL, lesions cleared spontaneously within 36 months. These findings, together with ours support the use of HCII in a cervical screening program. This has clinical importance, as a recent report from the US Preventive Services Task Force (USPSTF) [74] concluded that there was insufficient evidence to recommend HPV testing for cervical cancer screening. We noticed that the estimated accuracy of HCII (HSIL: sensitivity = 0.82, specificity = 0.78; LSIL: sensitivity = 0.66, specificity = 0.91) from the USPSTF report came from the 1999 study by Cuzick et al. [75] of older women. As Castle [76] pointed out, the conclusion from the USPSTF was reached without the results of randomized, controlled trials in Europe and India [60–62, 65], and HPV testing in the US was not evaluated. As more evidence accumulates for HPV testing, the results of our meta-analysis could be used as an additional tool for public health professionals as they decide the best test for their specific cervical screening programs.

The major strengths of our meta-analysis are the use of both the STARD [22] and MOOSE [23] reporting guidelines for study selection, data analysis, and comparison of test accuracy, which led to the important condition that both tests must be present in the same article. This eligibility criterion enabled us to minimize a substantial source of interstudy heterogeneity. The other strength is that our search allowed us to capture articles and studies from 1999 to the present, which is the time that HCII has been most widely used. The other strength is our inclusion of other important clinical outcomes that allows more conservative application of colposcopy.

The main limitation in our meta-analysis is that we did not include the technique/device for sample preparation (i.e., collection of cervical cells and DNA extraction methods) and age of the study participants, which could be two potential sources of interstudy heterogeneity. The large variety of sample collection and preparation methods (Table 1) prevented us from establishing meaningful groups for a categorical analysis. We also could not include the age variable in our meta-analysis because only two studies [31, 32] (Luu et al., submitted) provided the relevant information.

In summary, we found that while PCR is more sensitive but less specific than HCII in detecting ASCUS/LSIL, HCII has higher sensitivity and specificity than PCR in detecting HSIL, in both screening and diagnostic settings. Given the clinical relevance and importance of cervical cancer worldwide, our results support the use of HCII in cervical screening programs. Also the role of HPV type distribution should be explored to determine the worldwide comparability of HPV test accuracy. While cost of the test has a consideration for any screening program, it appears that the cost of both HCII and PCR has reduced overtime. Further studies on the cost-effectiveness of HCII over PCR in a cervical screening program are, therefore, warranted.
